# Temporal dynamics, population characterization and mycotoxins accumulation of *Fusarium graminearum* in Eastern China

**DOI:** 10.1038/srep36350

**Published:** 2016-11-17

**Authors:** Jian-bo Qiu, Jing-Tao Sun, Ming-Zheng Yu, Jian-Hong Xu, Jian-Rong Shi

**Affiliations:** 1 Key Lab of Food Quality and Safety of Jiangsu Province-State Key Laboratoriky Cultivation Base, Jiangsu Academy of Agricultural Sciences, 210014, China; 2 Key Laboratory of Control Technology and Standard for Agro-Product Quality and Safety, Ministry of Agriculture, Jiangsu Academy of Agricultural Sciences, 210014, China; 3 Collaborative Innovation Center for Modern Grain Circulation and Safety, Jiangsu Academy of Agricultural Sciences, 210014, China; 4 Institute of Food Quality and Safety, Jiangsu Academy of Agricultural Sciences, 210014, China; 5 Department of Entomology, Nanjing Agricultural University, Nanjing, 210095, China

## Abstract

Trichothecene genotype composition, mycotoxin production, genetic diversity, and population structure were analyzed, using 185 *Fusarium* strains collected from wheat (*Triticum aestivum* L.) throughout the Jiangsu province during 1976, 1983, 1998, 2006, and 2014. The results showed that 3-acetyldeoxynivalenol (3ADON) was consistently the predominant type in this region over 40 years, and the nivalenol (NIV) type has emerged since 1998. Long-term rotation of wheat and rice (*Oryza sativa* L.), rather than fungicide application, crop fitness, or weather conditions, might be the main cause of this phenomenon. The genetic diversity results from two toxin synthetic genes, *Pks4* and *Tri10*, and variable number of tandem repeat (VNTR) markers revealed the largest variance within the population in 1998, which was also the year with the highest production of mycotoxins. Population differentiation analysis indicated that major temporal population comparisons from the same area were not significantly differentiated. Our results showed that dominant species could maintain genetic stability for a long time, and *Pks4* would be of utility in genetic and population studies.

Fusarium head blight (FHB) is one of the most destructive and widespread diseases of wheat (*Triticum aestivum* L.), barley (*Hordeum vulgare* L.), and other cereals, globally. This disease reduces crop yields, causing extensive economic losses. More importantly, harvested grain quality is often compromised, due to mycotoxins produced by pathogens. Consumption of contaminated grains and products might have pernicious effects on human and animal health^1^.

*Fusarium graminearum* species complex (FGSC) consists of at least 16 phylogenetically distinct species, and is the main causal agent of FHB^2^,^3^,^4^,^5^,^6^,^7^. *Fusarium asiaticum* is the predominant species in Asia^8^,^9^,^10^,^11^,^12^, while *F. graminearum* sensu stricto (s. str.) is present in most FHB-occurring areas around the world, especially in America and Europe^3^,^4^,^6^. Members of FGSC produce toxic secondary metabolites, among which trichothecene and zearalenone (ZEN) are the most closely monitored due to their high detection rates and strong toxicity. Trichothecene toxins, including deoxynivalenol (DON) and nivalenol (NIV), inhibit eukaryotic protein biosynthesis and cause some human and animal mycotoxicoses^13^,^14^. FGSC strains have been shown to possess one of three trichothecene genotypes: 3-acetyldeoxynivalenol (3ADON), 15-acetyldeoxynivalenol (15ADON), or NIV^15^. Zearalenone (ZEN) and its metabolites have estrogen-like functions and strong teratogenic effects^16^. Moreover, ZEN has been shown to be genotoxic to cells and toxic to some animal tissues^17^. As a result, the mycotoxin-producing ability of individual isolates is always a concern.

With the development of molecular biology, sequences and functions of members in the trichothecene biosynthetic gene cluster have been gradually elucidated. The first gene discovered in the trichothecene pathway was *Tri5*^18^, which catalyzes the first reaction in this pathway. Thereafter, the other 11 core genes were subsequently discovered^19^. Although *Tri6* and *Tri10* are not located in that gene cluster, they were proven to be key regulators of synthetic genes^20^,^21^. The sequence basis for genotypic differences in trichothecenes has also been identified, and several specific primers have been developed to determine the trichothecene genotypes of individual isolates^22^,^23^,^24^,^25^,^26^,^27^. Although contradictory results about trichothecene genotypes and chemotypes were reported, these polymerase chain reaction (PCR)-based methods are important to determine trichothecene genotype distribution, and for large-scale molecular monitoring of FHB pathogen composition. More importantly, many key secondary metabolite biosynthetic genes in the *Tri* clusters have been used successfully in phylogenetic studies^28^. Zearalenone is synthesized through a polyketide pathway. Two different polyketide synthases, *PKS4* and *PKS13*, from the PKS gene cluster, are essential for ZEN production^29^,^30^. There is evidence that these two *PKS* genes are useful in phylogenetic studies as their intraspecific divergence^31^.

Genetic diversity and population structure, resulting from evolutionary forces over time and space, could be used to predict a pathogen’s response to disease management (e.g., fungicide application, crop rotation, and breeding for resistance). High genetic variation in a small region might suggest high adaptive capacity of the pathogen to different environments^32^. Population characterization of the FHB pathogen was conducted at various locations, based on restriction fragment length polymorphism (RFLP)^8^, random amplified polymorphic DNA^33^, sequence related amplified polymorphism (SRAP)^34^, amplified fragment length polymorphism (AFLP)^35^,^36^, and variable number of tandem repeats (VNTR)^37^,^38^. More recently, restriction site associated DNA sequencing (RADseq) was used in population genomics analyses of German populations^39^.

In China, the first recorded FHB severe outbreak was in 1936^40^, and after that FHB epidemics became more frequent and serious, especially since the beginning of the 21^st^ century. Scab was more severe throughout the Jiangsu province, due to the warm and humid climate during the flowering stage of wheat and other grains, which were heavily contaminated with high levels of mycotoxins. As a result, the *Fusarium* population dynamic genetic structure over the years, which reflects toxin-producing ability, merits further study.

In this study, a large number of samples, from five representative years, were used to investigate species composition and analyze the factors affecting FHB population dynamics. Genetic diversity and population structure were measured with VNTR markers and toxin biosynthetic genes, the effect of new molecular markers was assessed. We also attempted to explain the relationship between genetic diversity and mycotoxin accumulation.

## Results

### FHB pathogen species and trichothecene genotype composition

**Figure 1:**
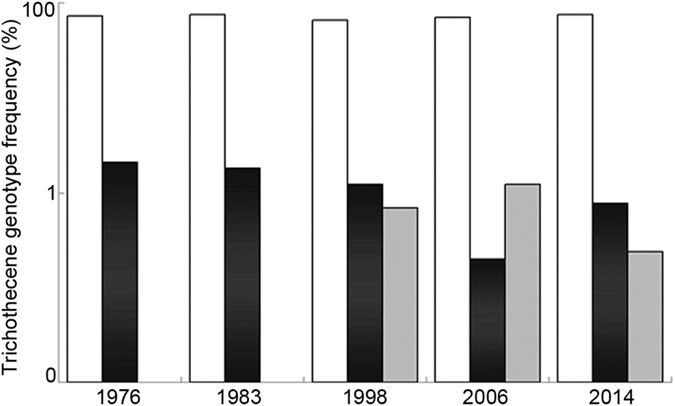
The frequency of 3-acetyldeoxynivalenol (3ADON) (white), 15-acetyldeoxynivalenol (15ADON) (black), and nivalenol (NIV) (gray) genotypes in each population.

Temporal trends of genotype frequency are shown in Fig. 1. The frequency of 3ADON *F. asiaticum* strains remained consistently high, always above 80%. *F. asiaticum* strains with the NIV genotype were not detected in the previous period. The NIV frequency was 8.33, 11.11, and 4.91% in 1998, 2006, and 2014, respectively. In *F. graminearum* s. str. strains, only 15ADON type was identified. The 15ADON ratio decreased between 1976 and 2014 (14.29–8.73%, *P* > 0.05; Fig. 1).

### Genetic diversity

**Figure 2:**
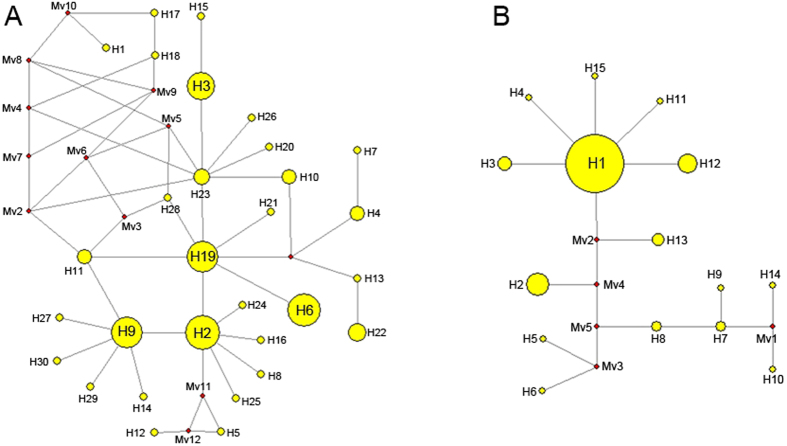
Haplotype network based on *PKS4* (**A**) and *Tri10* (**B**). The sizes of the yellow circles are in proportion to the frequencies of the haplotype. The red median vector is a hypothesized sequence, which is required to connect existing sequences within the network with maximum parsimony.

Partial sequences of two genes were amplified from all the samples. For *Pks4*, 55 polymorphic sites were detected, giving 30 haplotypes. Among them, 20 unique haplotypes existed in a single population, and the remainder were shared among populations. H2, H3, H6, H9, and H19 were the four largest haplotypes being all five populations and were found in 18, 16, 14 and 14 isolates, respectively (Fig. 2A). For *Tri10*, 98 nucleotides were variable among all samples. Fifteen haplotypes were confirmed, of which eight were unique and seven were present between populations. H1, the largest haplotype, was found in 69 individuals among five populations (Fig. 2B).

Basic genetic diversity indices calculated for *Pks4* and *Tri10* are listed in Table 1. There is high haplotype diversity and low nucleotide diversity for the *Pks4* and *Tri10* sequences, especially for the former. Overall haplotype and nucleotide diversity for *Pks4* were 0.912 and 0.501%, respectively. Overall haplotype and nucleotide diversity for *Tri10* were 0.569 and 1.617%, respectively. The population F1998 showed the largest numbers of haplotypes (*N*_h_) and the greatest haplotype diversity (*H*_d_; Table 1).

The eight VNTR markers used for this study were highly polymorphic, and the numbers of alleles in each locus varied from 5 to 18. However, the number of effective alleles in each population was relatively low, with a mean of 6.086. Highly unbiased gene diversity was observed in all populations, and ranged from 0.561 to 0.683. In general, F1998 was found to have higher values of alleles and greater gene diversity (Table 1).

### Population structure

**Figure 3:**
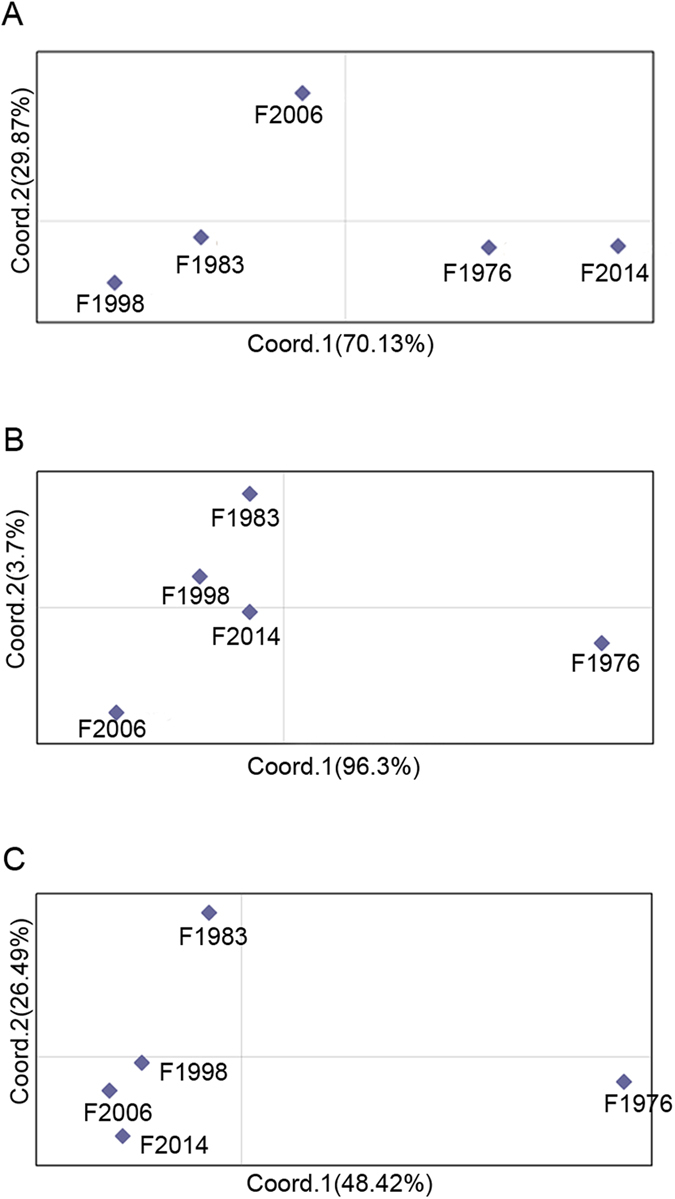
Principal coordinates analysis on pairwise *F*st values at *Tri10* (**A**), *pks4* (**B**) and variable number of tandem repeat (VNTR) markers (**C**).

Table 2 displays pairwise *F*st values from *Pks4* and *Tri10* sequences. For *Tri10*, haplotype frequencies among years were not significantly different. Exact tests showed that significant differences were present in 4 of the 10 pairwise populations at *Pks4*. The *P* values were 0.006 for F1976 vs. F1983, 0.012 for F1976 vs F1998, 0.025 for F1976 vs. F2014, and 0.011 for F1983 vs. F2006. The principal coordinate analysis (PCA) results showed that five populations had minimal genetic structure at *Tri10*, while F1976 was separated from the other populations for *Pks4* (Fig. 3).

The unbiased genetic distance was low and genetic identity was high among the groups, based on VNTR markers (Table 3). In addition, low genetic differentiation and high gene flow were observed, indicating that a low level of genetic differentiation was present in *F. asiaticum* populations, and spatial variation had a minimal effect on population subdivision in this region (Table 4). Significant differences in *F*st values were present in F1976, compared to the other four populations, and F1976 was independent of those populations (Fig. 3). VNTR data were similar to the data from *Pks4.*

### Fitness

Colony diameter and conidia production are listed in Table 5. There were no obvious differences in growth rates among the isolates of the five populations, although several isolates grew slowly because of long-term preservation. *In vitro* analyses of toxin production for most isolates confirmed the accuracy of the chemotype detection by PCR, and indicated that mycotoxin accumulation of the isolates from F1998 was significantly (*P* < 0.05) higher compared to that of the remaining groups. Pairwise comparisons of this indices among the other four populations showed no obvious difference.

### Weather conditions

**Figure 4:**
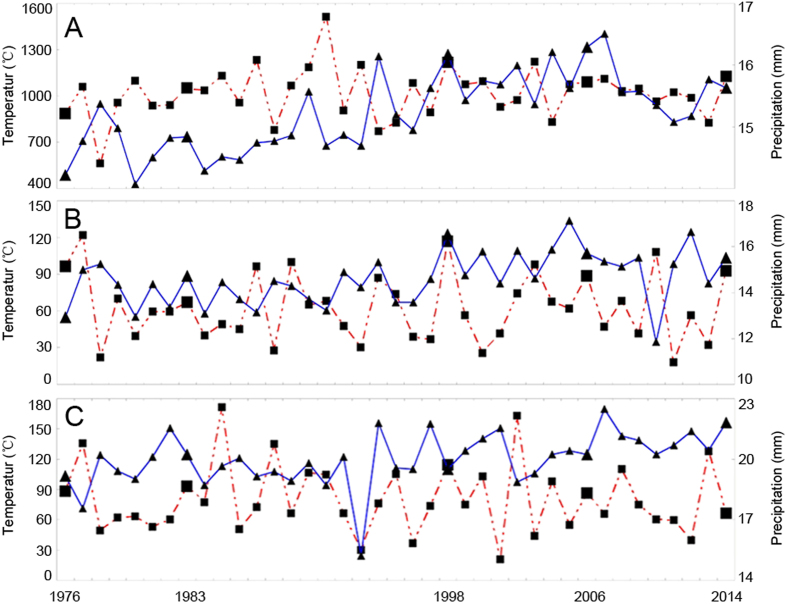
Average temperature and precipitation time courses in Jiangsu province between 1976 and 2014. Weather data from 24 meteorological stations distributed over this region were averaged. Temperature data (blue solid line) represent averages for the whole year (**A**), April (**B**), and May (**C**). Precipitation data (red dashed line) represent average amounts for the whole year (**A**), April (**B**), and May (**C**). The large triangles and squares are used to indicate key years.

The yearly annual rainfall has fluctuated in the past 40 years, ranging from 560 to 1,510 mm. The mean temperature ranged between 14 to 16.5 °C in the same period. Large fluctuations in these two factors were observed, especially in adjacent years (Fig. 4). Both factors initially increased, and then were reduced during the 5-years study period, with the highest precipitation amount and air temperature being in 1998 and 2006, respectively (Table 6).

A similar trend was observed at the anthesis stage, especially in April, and two meteorological parameters were at their highest in 1998. In May, rainfall was significantly higher in 1998. This region was particularly cold in 1998 and the highest temperature was recorded in 2014 (Table 6).

## Discussion

### Factors involved in population composition

The FHB pathogen composition and population dynamics have been studied widely and intensively in North America and China. Ward *et al.*^37^ found significant increases in 3ADON frequency, between 1998 and 2004, in the western Canadian provinces, including Manitoba and Saskatchewan^37^. Similar temporal trends were reported for the midwestern United States and Canada^41^,^42^,^43^. Contradictory results have also reported. There were no significant differences in the 3ADON ratios in Alberta, of 1998 vs. 2004 and 2005 vs. 2007, which was not the case in Manitoba and Saskatchewan^43^.

In China, the 3ADON ratio of the *F. asiaticum* strains in the middle valley of the Yangtze River increased from 53% in 1999 to 87% in 2006^12^,^44^. However, it should be noted that the limited numbers of strains, collected from only a few counties, might not be representative of large provinces.

Concerning the Jiangsu and Anhui provinces specifically, the frequency of the *F. asiaticum* strains was 74.12% in 1999 and 85.71% in 2006, similar to the results obtained in the previous study. We previously analyzed a large number of FHB isolates in the Jiangsu and Anhui provinces in this region in 2011 and 2012, and discovered a slight, but non-significant, decrease in 3ADON production^38^. In the present study, an expanded analysis of the FHB pathogen was conducted during the past 40 years, and no obvious differences were found in the population dynamics. These results suggest that the *Fusarium* population follows a unique evolutionary trajectory in a diverse region. The bottle-neck effect, or certain other factors, might cause this phenomenon and should be considered. Continuous monitoring of the FHB pathogen in multiple areas, and collection of related data, might shed more light on these results.

There is no clear evidence to explain the chemotype dynamics. A number of previous studies indicated that the composition of chemotypes appeared to be phylogenetic species-dependent. In Asia, NIV and 3ADON are produced by *F. asiaticum*, while *F. graminearum* isolates accumulate 15ADON^8^,^9^,^10^,^11^,^12^.

The FHB pathogen population might be adapted to different plants^12^. A shift in pathogen population occur according to crop rotation and host. The Jiangsu province has a long history of rice growing, in an area covering 30 million acres. In contrast, about 10% of the wheat harvesting area is under rainfed crops cultivation; this applies to maize (*Zea mays* L.), cotton (*Gossypium* spp. L.), and soybean (*Glycine max* L.). Extensive wheat-rice rotation is critical for *F. asiaticum* overwintering, and perithecium production typically favors rice straw under warmer conditions. As a result, a 3ADON-producing *F. asiaticum* population could be present within this region for a long time, and we suggest this might have been the main factor underlying the absence of variation in trichothecene genotype frequencies from 1976 to 2014. Recently, more *F. asiaticum* isolates with the 3ADON genotype and DON content were detected in wheat samples when rice was the preceding crop (unpublished data). Many publications have also reported a profound influence of host prevalence on *F. graminearum* species and trichothecene chemotype composition^9^,^12^. Kilx *et al.*^45^ indicated that previous crops affected variation in DON content, although there was no impact on species composition, as it could only influence primary sources of infection for all species.

Environmental factors were closely correlated with FHB outbreak and the distributions of FHB pathogens, especially at anthesis^46^. Backhouse^47^ developed a model (BIOCLIM) to quantify the differences in the climatic range of FGSC members and indicated that *F. asiaticum* occurs in warm (mean temperature >22 °C) and wet (rainfall >320 mm) areas, while *F. graminearum* is not limited by climatic conditions. The average annual temperature and rainfall data of our region showed remarkable variation during the past 40 years, particularly in consecutive years. The average temperatures were between 13.5 and 16 °C, which seemed to be lower than the results from modeling. In a previous study, 15 °C was recommended as the boundary between *F. asiaticum* and *F. graminearum*^48^, with this being more applicable in China. Mean precipitation was higher than 320 mm in every year and mainly occurred during anthesis. Abundant rainfall and warm temperatures provide a superior environment for *F. asiaticum.*

Yang *et al.*^49^ assessed the relationship between 3ADON chemotype shifts in the Yangtze River valley and climatic conditions, but found no significant association. Although there were annual temperature differences between certain years in the current study, we also observed a similar composition of populations. *Fusarium* species differed in their responses to air temperature and humidity, as mentioned in several previous reports. Low rainfall and relative humidity during the anthesis stage caused a lower number of 15ADON-producing *F. graminearum* strains, which were replaced in *F. avenaceum* with the 3ADON chemotype, and in *F. poae* with the NIV chemotype, in Luxembourg^50^. A similar result was reported in the Italian population^51^. Pathogenic and environmental factors involve a long process of mutual adaptation; therefore, fluctuations in weather conditions over short time periods are unlikely to have a large effect. More rigorous, longer-term research should be based on detailed information regarding environmental factors and the composition of *Fusarium* species.

Winter wheat cultivars, grown throughout the Jiangsu province between 1976 and 2014, varied over time. The planting areas for those varieties showed more resistance toward *Fusarium* species. This resistance continued to increase over the period, suggesting minor effects of long-term changes in cultivar composition and species shifts. Low-level genetic differentiation, and the distances observed between the resistant and susceptible wheat populations in the US, proved that FHB cultivars have little effect on *F. graminearum* population division^52^. Meanwhile, there were no major changes in the fungicides applied for FHB, as carbendazim has been used to control this disease over the past four decades. Phenamacril and tebuconazole have high activity against *Fusarium* species and favorable effects on FHB. Long-term single fungicide application could only induce decreased sensitivity, but no changes in pathogen structure.

Population genetics analysis in a previous study revealed that two populations were genetically similar, based on the sensitivity to carbendazim^38^. No significant differences in the frequency of isolates with DON or NIV markers were observed between the two groups. In addition, *F. asiaticum* strains were found to be more receptive to carbendazim under selection pressure, which could partially explain the predominance of 3ADON-producing *F. asiaticum* populations. Similarly, carbendazim resistance was only detected in *F. asiaticum* groups with 3ADON or NIV genotypes, from 2010 to 2012^53^.

Ward *et al.*^37^ found that 3ADON populations with greater toxin accumulation, higher growth rates, and greater conidia production replaced the 15ADON population, suggesting that fitness is an important factor affecting FHB population shifts. Additional similar data indicated that these factors give 3ADON strains a direct fitness advantage and drive the rapid shift in *Fusarium* composition^41^,^54^. However, these studies focused principally on pathogenic traits. Spolti *et al.*^55^ compared 14 attributes of saprophytic and pathogenic fitness, between *F. graminearum* strains of 3ADON and 15ADON genotypes, and found no differences. This further suggested that it was the result of recombination and natural selection; through which genes conferring phenotypic advantages might be present in any trichothecene genotypes.

There were no clear distinctions in mycelial growth or spore production among the five populations in our study. Although DON and ZEN levels varied, there were no significant effects on pathogen dynamics, as species composition remained stable. As 15ADON isolates were rare in all populations, we could not compare these attributes between two groups with different genotypes. Individual fitness might vary, and those isolates with advantages expand faster and dominate during evolution. It is possible that 3ADON populations replaced the others much earlier, as single 3ADON groups were predominately present in this region from the 1970s.

### Mycotoxin accumulation and genetic diversity

The development of FHB, and accumulation of mycotoxins in infected wheat grains, are influenced by many factors from pre-sowing to harvest. We attempted to determine the causes of the relatively consistent presence of the FHB pathogen and found significant differences in rainfall for certain years. The heaviest rainfall occurred in 1998, and the highest levels of mycotoxins were detected in the population F1998. Based on these results, we speculate that rainfall at anthesis or throughout the year promoted mycotoxin productivity of most isolates in 1998. Unfortunately, we have no data on the mycotoxin contents in grains from 1976, 1983, and 1998. In a previous article, we found a clear relationship between higher precipitation and high levels of DON and ZEN^56^. Some recent publications have suggested that environmental conditions, especially moisture in the form of rainfall or relative humidity, are associated with higher DON and ZEN accumulation^50^,^57^,^58^.

Another factor worthy of attention is the application of carbendazim, which is effective for controlling FHB and toxin accumulation^59^. This fungicide was introduced to control FHB during the 1980s in China, and the first carbendazim-resistant *F. graminearum* was detected in 1992^60^. We found a similar trend, in which the sensitivity of *Fusarium* strains to carbendazim gradually decreases with frequent use. This could partially explain the increased toxin production levels of *Fusarium* isolates from 1973 to 1998. The fact that the largest amount of toxins was in F1998 might be due to resistance to carbendazim, which could increase trichothecene production *in vivo* or *in vitro*. Similar phenomena have been reported, attracting attention since 2000, when more efficient fungicides, including tebuconazole and phenamacril, were introduced^61^. Integrated management practices were also developed to prevent FHB, as mycotoxins have become a more prominent issue. These methods could reduce toxin contamination in grains, at least to a certain degree.

The population F1998, which has the maximum genetic diversity, produced the largest amounts of mycotoxins as fitness-related traits. Extensive studies focusing on the relationship between genetic variability and population fitness, or fitness-related characteristics, have been conducted by evolutionary and conservation biologists^62^,^63^. It has become gradually accepted that populations, particularly of plants and animals, with low genetic diversity values show reduced fitness, even in optimal environments.

In the present study, some fitness-related traits were compared between populations and no differences were found in terms of mycelium growth or spore yields. These basic abilities ensure survival and reproduction of individual isolates. However, those populations with high genetic diversity or a high number of haplotypes showed increased capability in terms of toxin synthesis. Many published papers have indicated that low genetic diversity could induce inbreeding depression effects, while high genetic diversity increased population fitness, as mediated by heterosis^63^,^64^. We should be more concerned with low genetic diversity in terms of the capacity of a population to adapt to a novel environment, especially a stressful one. We analyzed fitness traits and genetic diversity, and further studies focusing on these two factors merit are merited.

Varying levels of genetic diversity of *F. asiaticum* populations in China were described previously, with groups from southwestern locations harboring the greatest diversity^12^,^65^. Elevation and mountains might hinder genetic exchange and external genotypes^49^,^65^. In terms of the results from several relevant research articles on VNTR markers, the gene diversity values (0.5–0.6) in the present study were in the normal range. Ward *et al.*^37^ indicated that eastern Canada was the origin of the 3ADON population in North America, and that 3ADON isolates spread across Canada and into the US. In China, dozens of 3ADON-producing strains were isolated as early as 1976. The results of multi-year molecular surveillance demonstrated that 3ADON-producing *F. asiaticum* strains have been present within this region for at least 40 years. Yang *et al.*^49^ considered NIV-producing *F. asiaticum* isolates in the mountain-rich provinces to represent the older dominant populations in China, which have been replaced by DON producers. Zhang *et al.*^12^ confirmed this hypothesis by also conducting genotyping analysis, and determined the migration direction of *F. asiaticum*. Regional studies should be conducted to understand the spread of the 3ADON population.

Mycotoxin production of individual strains varied widely, even in the same population, and the process was under gene regulation. Some nucleotides of mutations were detected in high toxin-producing strains in two genes. Further study is required to determine if these mutations could influence a single individual’s ability to regulate the synthesis of secondary metabolism.

### Population differentiation

The VNTR data revealed significant population subdivision between F1976 and the subsequent population. High gene flow and low population differentiation in the latter groups indicated that individual temporal populations were part of a single metapopulation, at least from 1983 to 2014. Several studies focusing on *F. asiaticum* or *F. graminearum* populations from North America and East Asia reported a similar genetic structure, suggesting a pathogen population from a small, isolated disease nursery might remain stable for a certain period of time^8^,^35^,^66^,^67^. In a previous study, using the isolates from two consecutive years, we also found no subdivisions^38^. However, population subdivisions based on the trichothecene type, geographic differences, and temperature gradient from a larger geographic area, have been reported^6^,^12^,^15^,^68^. Long-term exclusive trichothecene genotypes had no effect on population differentiation in this region. The reason for the significant divergence between the original group and the succeeding groups is unknown. By considering the similar geography, weather conditions, trichothecene genotypes, and biological traits of these populations, we will continue to study the effects of longer time periods on the population structure of this pathogen.

The VNTR markers developed for FGSC have been successfully applied to population analyses in recent years. In this study, we used amplified fragments to characterize population differentiation and acquired divergence between *Pks4* and *Tri10*. Genetic diversity based on those two genes was coincidental, although haplotype diversity values were higher for *Pks4* than for *Tri10* in the same population. There were inconsistent results regarding population differentiation by these two genes. At *Tri10*, population differentiation values were low and non-significant between each pair. At *Pks4*, F1976 was demonstrated to be significantly separated from the other populations, and this finding was very similar to that for the VNTR data. According to our results, some specific genes with rich nucleotide polymorphisms could be used in genetic and population characterization studies. We also tried to introduce *Tri6* and *Pks13*, but failed to acquire long and single fragments in preliminary amplification; this work is continuing.

Inconsonant genetic results pertaining to *Fusarium* populations with two methods are rare. Ouellet and Seifert (1993)^69^ reported similar results with random amplified polymorphic DNA (RAPD) and PCR. Recently, Talas and McDonald (2015)^39^ utilized restriction site-associated DNA sequencing (RADseq) to analyze population structures in German *F. graminearum* populations, and detected low population differentiation. In an earlier study on the same German isolates, these authors found higher *F*st values with VNTR markers^70^. Varied results might be drawn according to different types, numbers, or distributions of molecular markers across the genome; adoption of more markers could lead to more reliable conclusions.

## Methods

### Isolate collection

In total, 185 isolates were used for this study, including 26 collected in 1976, 28 collected in 1983, 35 collected in 1998, 46 collected in 2006, and 50 collected in 2014, from different counties in the Jiangsu province of eastern China. Those isolates were grouped according to the sampling time (F1976, F1983, F1998, F2006, and F2014). The first three populations were stored in 20% glycerol at −80 °C for long-term storage. The other isolates were obtained from diseased wheat grain, following methods developed previously^38^.

### Species and trichothecene genotype identification

For DNA extraction, mycelia, harvested from single-spore cultures on potato dextrose agar medium (PDA), was ground by drill and then pulverized with lysis buffer (Tris-HCl 200 mM, EDTA 50 mM, NaCl 20 mM, SDS 1%). The mixture was centrifuged at 12000rpm for 10 min and the supernatant was mixed with an equal volume of absolute ethanol at −20 °C for about 2 h. The DNA was precipitated after another centrifugation. After a wash with 70% ethanol, pure DNA was air dried and dissolved with sterilized water.

Fg16F⁄R primers that produce polymorphic products (400–500 bp) with DNA from members of the *F. graminearum* species complex were used to identify each isolate by species. The 410-bp DNA fragment was amplified from the isolates belonging to *F. graminearum*, while the 497-bp fragment was generated from the isolates of *F. asiaticum*^48^,^71^. Chemotypes of the FGSC isolates were determined using the specific primer pair described by Li *et al.*^25^. Another two primer sets, Tri303F/Tri303R and Tri315F/Tri315R, which target the *Tri3* gene^23^, were used to further characterize DON chemotypes of the *F. graminearum* complex, as 3ADON or 15ADON.

### VNTR analysis

Eight VNTR markers, developed by Suga *et al.*^72^, were used to evaluate the population structure and diversity of all *F. asiaticum* strains. The forward primer of each marker was labeled with either fluorescent 6-FAM (HK630, HK1043, HK1059, and HK957) or PEX (HK917, HK1073, HK913, and HK967) and PCR was performed as described previously^72^. GENALEX 6.5^73^ was used to analyze pairwise gene flow (*Nm*), population differentiation or fixation index (*Fst*), allele frequencies, unbiased gene diversity (*H*), Nei’s unbiased genetic distance, and Nei’s unbiased genetic identity.

#### Sequence analysis

Portions of the *Pks4* gene (1944 bp), and the complete *Tri10* gene (1194 bp) of all *F. asiaticum* isolates, were amplified by PCR. The primer pairs for *Pks4* (F: CTGGTCTCTTGGACACGCTT; R: TTCAGTTGCTTTCGCCTTGC) and *Tri10* (F: CGAGAGACGAGCCTGTTGAT; R: GCTGAATGTCGCCTCATACG) were designed based upon the genome sequence of *F. graminearum* PH-1 (http://www.broad.mit.edu). PCR was performed in 40 μL reaction volumes with 10 ng of template DNA, 0.3 μL of Taq polymerase, 2.5 μL of Taq Buffer, 0.5 μL of dNTP, and 1 μL of each primer (10 mM). The amplification profile consisted of an initial denaturation of 94 °C for 3 min, followed by 35 cycles of denaturing (30 s at 94 °C, 40 s at 58 °C, 40 s at 72 °C) and an additional extension at 72 °C for 10 min. PCR-amplified DNA fragments were excised from the gel and purified using an AxyPrep DNA Gel Extraction Kit (Axygen, Hangzhou, China) according to the manufacturer’s instructions and sequenced by Shanghai Shenggong Biotechnological Ltd.

DNA sequences were aligned and edited using the MEGA software package, and the truncated sequences of the two genes were then concatenated into a data matrix for subsequent analysis. To assess the genetic variation within populations, the number of haplotypes (*N*_h_), nucleotide diversity (π), and haplotype diversity (*H*d) were calculated with DnaSP software (ver. 5). The degree of population differentiation was quantified using pairwise *F*_ST_ values in Arlequin (ver. 3.5). Genotypic differentiation between pairwise populations was detected by an exact test. To visualize these genetic relationships between populations, the pairwise *F*_ST_ matrix was then used to perform PCA in GenAlEx software (ver. 6.5)^73^. The distribution of haplotypes was represented, first through a median joining network constructed with Network software (ver.4.6).

### Mycotoxin content analysis

Mycotoxin production of *Fusarium* isolates was assessed using a previously described method^56^, with minor modifications. The autoclaved diced substrate (20 g) was inoculated with 20 agar plugs of each isolate and stored in the dark for 30 d at 25 °C. Three replicate units were prepared for each isolate. Colonized wheat cultures were pulverized and 5 g samples were used for mycotoxin extraction and quantification.

### Growth rate and sporulation capacity

Mycelial plugs (5 mm in diameter) from the edge of a 3-day-old colony of each strain were placed on PDA plates at 25 °C. Radial growth was measured after 3 d. Ten plugs, as described above, were added to a conical flask containing 100 mL liquid CMC, and the flask was placed on a shaker for 7 d (150 rpm, 25 °C, 12 h photoperiod). Spore production was determined with a hemacytometer. Three replicates were used for each strain.

### Meteorological data

Yearly rainfall and average temperature records from available meteorological stations throughout the Jiangsu province, from 1976 to 2014, were downloaded from a Chinese meteorological data service system (http://data.cma.gov.cn). Climatic data during wheat anthesis (April and May) were also obtained at 24 observation stations, as listed in Table S1.

## Additional Information

**How to cite this article**: Qiu, J.-b. *et al.* Temporal dynamics, population characterization and mycotoxins accumulation of *Fusarium graminearum* in Eastern China. *Sci. Rep.* **6**, 36350; doi: 10.1038/srep36350 (2016).

**Publisher’s note**: Springer Nature remains neutral with regard to jurisdictional claims in published maps and institutional affiliations.

## Supplementary Material

Supplementary Information

## Figures and Tables

**Table 1:** Basic genetic diversity indices calculated for *Pks4*, *Tri10*, and VNTR markers in *Fusarium asiaticum* populations.

Population	*Pks4*			*Tri10*			VNTR			
	*N*_h_	*H*_d_	π (%)	*N*_h_	*H*_d_	π (%)	*N*_a_	*N*_e_	*I*	u*H*
F1976	7	0.846	0.731	3	0.589	1.041	3.857	2.555	1.015	0.561
F1983	8	0.891	0.626	7	0.622	1.981	5.286	3.436	1.303	0.658
F1998	15	0.931	0.533	8	0.692	2.329	7.429	3.711	1.473	0.683
F2006	12	0.871	0.311	7	0.537	1.251	6.714	3.009	1.27	0.616
F2014	12	0.887	0.493	3	0.475	0.831	7.143	2.867	1.223	0.593
Total	30	0.912	0.501	15	0.569	1.617	6.086	3.116	1.257	0.622

VNTR, variable number of tandem repeats; *N*_h_, number of haplotypes; *H*_d_, haplotype diversity; π, nucleotide diversity; *N*_a_, number of alleles; *N*_e_, number of effective alleles; *I*, Shannon’s information index; u*H*, unbiased gene diversity.

**Table 2:** Pairwise comparisons of genetic differentiation of *Fusarium asiaticum* populations, based on *Pks4* (above diagonal) and *Tri10* (below diagonal).

Population	F1976	F1983	F1998	F2006	F2014
F1976	…	0.039**	0.044	0.076**	0.034**
F1983	−0.022	…	−0.024	0.008**	−0.011
F1998	0.004	−0.028		−0.015	−0.016
F2006	0.005	−0.001	0.031		−0.004
F2014	−0.051	0.019	0.042	0.028	…

**Table 3:** Pairwise comparisons of unbiased genetic identity (above diagonal) and genetic distance (below diagonal) of *Fusarium asiaticum* populations based on VNTR markers.

Population	F1976	F1983	F1998	F2006	F2014
F1976		0.927	0.921	0.912	0.908
F1983	0.076		0.982	0.982	0.957
F1998	0.082	0.018		0.999	0.976
F2006	0.092	0.018	0.001		0.999
F2014	0.097	0.044	0.025	0.001	

**Table 4:** Pairwise comparisons of gene flow (*Nm*, above diagonal) and genetic differentiation (below diagonal) of *Fusarium asiaticum* populations based on VNTR markers.

Population	F1976	F1983	F1998	F2006	F2014
F1976	…	9.806	9.344	8.171	7.462
F1983	0.049**	…	54.542	42.791	17.145
F1998	0.051**	0.009		151.603	26.551
F2006	0.058**	0.012	0.003		−0.004
F2014	0.063**	0.028	0.018**	−0.002	…

^**^Indicates significant difference (*P* < 0.05).

**Table 5:** Mycotoxin production, mycelial growth, and conidia production of *Fusarium asiaticum* populations.

Population	DON (mg/kg)	3ADON (mg/kg)	15ADON (mg/kg)	ZEN (mg/kg)	Mycelial growth (mm)	Sporulation capacity ( × 10^6^)
F1976	55.71 ± 45.82b	2.06 ± 1.15b	1.37 ± 1.33a	0.29 ± 0.21b	62.52 ± 10.16a	1.96 ± 2.02a
F1983	70.23 ± 69.39a	4.87 ± 4.19b	1.83 ± 1.04a	0.76 ± 0.69a	62.73 ± 14.69a	1.43 ± 1.37a
F1998	84.87 ± 71.27a	6.94 ± 9.62a	2.64 ± 3.06a	0.91 ± 0.78a	63.54 ± 12.71a	2.08 ± 2.31a
F2006	46.14 ± 38.55b	7.47 ± 9.14a	2.47 ± 3.04a	0.27 ± 0.38b	63.48 ± 11.11a	1.06 ± 1.08a
F2014	37.01 ± 49.81c	1.04 ± 2.31c	1.673.97a	0.12 ± 0.28	69.92 ± 7.55a	1.66 ± 1.54a

DON, deoxynivalenol; 3ADON, 3-acetyldeoxynivalenol; 15ADON, 15-acetyldeoxynivalenol; ZEN, zearalenone.

Means followed by different letters in each column are significantly different between populations according to the least significant difference (LSD) test at *P* = 0.05.

**Table 6:** Average temperature and precipitation amounts in April, May, and for the whole year for five different years.

Year	April		May		Full-year	
	Temperature (°C)	Precipitation (mm)	Temperature (°C)	Precipitation (mm)	Temperature (°C)	Precipitation (mm)
1976	12.923 ± 1.086d	96.392 ± 34.736a	19.169 ± 1.065c	88.038 ± 24.56b	14.219 ± 0.768d	886.692 ± 178.524c
1983	14.723 ± 0.745c	67.1 ± 41.576b	20.223 ± 1.007b	92.785 ± 49.222b	14.844 ± 0.601c	1051 ± 187.251b
1998	16.523 ± 1.329a	116.9 ± 37.305a	19.492 ± 1.029c	114.615 ± 54.522a	16.171 ± 0.789a	1209.362 ± 141.408a
2006	15.707 ± 1.16b	88.531 ± 28.786b	20.223 ± 0.859b	86.469 ± 26.997b	16.289 ± 0.799a	1089.6 ± 103.179b
2014	15.515 ± 0.733b	92.674 ± 43.543b	21.754 ± 0.724a	66.148 ± 18.468c	15.63 ± 0.864b	1127.465 ± 203.944b
