# Supplementary material for: Temporal dynamics, population characterization and mycotoxins accumulation of Fusarium graminearum in Eastern China
Source: Sci Rep. 2016 Nov 17;6:36350. doi: 10.1038/srep36350 (PMC5113074; doi:10.1038/srep36350)
Supplement: Supplementary Information [file srep36350-s1.pdf]

Temporal dynamics, population characterization and mycotoxins accumulation of *Fusarium graminearum* in Eastern China

Jian-bo Qiu[1], Jing-Tao Sun[2], Ming-Zheng Yu[1], Jian-Hong Xu[1,#], Jian-Rong Shi[1,#]

[1]Key Lab of Food Quality and Safety of Jiangsu Province-State Key Laboratory Cultivation Base; Key Laboratory of Control Technology and Standard for Agro-Product Quality and Safety, Ministry of Agriculture; Collaborative Innovation Center for Modern Grain Circulation and Safety; Institute of Food Quality and Safety, Jiangsu Academy of Agricultural Sciences, 210014, China.

[2]Department of Entomology, Nanjing Agricultural University, Nanjing, 210095, China

[#]Corresponding author: xujianhongnj@126.com; shiji@jaas.ac.cn

Table S1.24 observation stations in Jiangsu province used in this text.

No.	Observation stations
1	Pizhou
2	Xuzhou
3	Shuyang
4	Ganyu
5	Guanyun
6	Suining
7	Sihong
8	Xuyi
9	Huaian
10	Funing
11	Huaiyin
12	Sheyang
13	Dafeng
14	Nanjing
15	Gaoyou
16	Dongtai
17	Rugao
18	Nantong
19	Lvsi
20	Changzhou
21	Liyang
22	Wuxi
23	Kunshan
24	Dongshan
